# *Neisseria meningitides* Can Survive in Corpses for At Least Eleven Days

**DOI:** 10.3389/fcimb.2016.00074

**Published:** 2016-07-13

**Authors:** Antonio Osculati, Silvia D. Visonà, Alberto Colombo, Petra Basso, Luisa Andrello, Antonio Toniolo

**Affiliations:** ^1^Department of Public Health, Experimental and Forensic Medicine, University of PaviaPavia, Italy; ^2^Laboratory of Microbiology, ASST-Settelaghi, University of InsubriaVarese, Italy; ^3^Department of Biotechnology and Life Sciences, University of InsubriaVarese, Italy; ^4^Service of Legal Medicine of Canton TicinoBellinzona, Switzerland

**Keywords:** *Neisseria meningitidis*, survival, meningococcal sepsis, forensic autopsy, infectious disease transmission

Among the potential hazards of working in a mortuary and handling corpses, the risk of infectious disease acquisition is well-documented, and warrants attention. The main biological risk in this environment is due to infections caused by *Mycobacterium tuberculosis*, blood-borne hepatitis, and agents responsible for transmissible spongiform encephalopathies, such as variant Creutzfeld-Jakob disease. All these pathogens remain alive and are infectious postmortem. In addition, other pathogens present in cadavers, such as *Neisseria meningitidis*, are a potential source of infection during necropsy (Burton, 2003).

Generally, it is believed that pathogens do not survive for more than a few minutes on environmental surfaces; however, *N. meningitidis* has been found to survive for up to 72 h on glass and metal surfaces (Tzeng et al., 2014). To date, there is a lack of data regarding the duration of survival of *N. meningitidis* in corpses, even though people who handle cadavers are commonly considered to be at risk of infection (Burton, 2003).

During forensic examinations, deaths due to meningitis are often encountered. Recently, *N. meningitides* was detected in two corpses preserved at 4°C for 11 and 7 days, respectively, at our hospital. The first and more remarkable case regards a 28-year-old woman who died from fulminant meningitis. The anamnesis was negative until the day before she died. The woman presented to the Emergency Department with a high fever, shivers, nausea, and vomiting. The objective examination did not indicate any significant findings, except for abdominal tenderness and inflammation of the pharynx. Therefore, the patient was discharged. A few hours later, the woman returned to the same hospital with a higher fever (40°C) and persistent vomiting. Then, she was admitted to the Department of Internal Medicine. At the time of admission, blood samples were taken for microbiological testing, and antibiotic therapy (3 g of ampicillin and 2 g of cefotaxime) was administered. The clinical exams did not find anything remarkable except for the fever. At 2 h after admission, the woman presented with breathing difficulty, hypotension, diffuse petechiae, mild rigor nuchalis, and a temperature of 37.5°C.

The patient was intubated, mechanical ventilation was enacted, and then she was brought to the Intensive Care Unit, where, 2 h later, she died. The microbiological test results, available after death, pointed out the presence of group C *N. meningitidis* in the blood. The day after, a medical autopsy was performed: macroscopically, diffuse petechiae and massive hemorrhage of the adrenal glands were observed. Bacterial cultures of the blood samples detected the growth of group C *N. meningitidis*.

Eleven days after the woman's death, the prosecutor ordered an additional autopsy in order to investigate the hypothesis of medical malpractice. Before the internal examination, using a carefully sterile technique, bacteriological samples were taken from blood (particularly from the neck vessels. Then, during the internal examination, samples of brain, liver, adrenal gland, bone marrow were collected using sterile instruments and containers.

Briefly, blood (100 μL) was plated on selective Martin Lewis Agar (Becton Dickinson, Heidelberg, DE) and incubated in aerobic environment with 5% CO_2_ for 48 h.

Specimens of other tissues were first inoculated in Brain Heart infusion Broth (Becton Dickinson, Sparks, US) and incubated in same conditions. After 24 h, 100 μL of broth were plated on Martin Lewis Agar and incubated in conditions of aerobic environment with 5% CO_2_.

After 48 h of incubation we revealed growth of colonies on all the culture plates: in particular, on the plate inoculated with blood sample, we found more than 10^6^ Colony Forming Units (CFU).

At gram staining they appeared as Gram negative diplococci, with positive reaction for oxidase and catalase, suggesting *Neisseria* spp.

The bacteria were identified as *N. meningitidis* using MALDI-TOF MS (Bruker Daltonics, Germany). Slide agglutination (Remel, US) showed reaction with serum against capsule polysaccharide of serogroup C.

At the same time, we performed a molecular analysis on all the specimens.

DNA extraction from clinical samples was carried out using the QIAamp DNA Mini Kit (Qiagen), according to manufacturer instructions. Purified DNA were kept at −80°C.

Bacterial DNA was amplified both with 16S rRNA primers, a primer pair targeting ctrA gene for identification of *N. meningitidis* species, and six primers pairs targeting serogroup-specific capsule biosynthesis genes (A, B, C, X, Y, W135) of *N. meningitidis*.

PCR was run with 35 cycles of 95°C for 30 s, 60°C for 1 min, 72°C for 1 min, and a final extension of 72°C for 10 min. A 10 μl of amplified product was run in a 1% agarose gel stained with ethidium bromide. Amplified products were visualized and photographed under UV light.

PCR analysis confirmed identification of *Neisseria meningitidis* Group C.

These results allowed us to confirm that the woman's death was due to fulminant sepsis from group C *N. meningitidis*, as suggested by the clinical and premortem microbiological exams. More remarkably, these findings pointed out that the pathogen was still alive in various organs of the corpse at 11 days after death and able to grow in culture.

The other case concerns a 6-month-old girl who was brought to the Emergency Department with a high fever (up to 39°C). A few hours later, the baby presented with cyanosis and breathing difficulty, and then she suddenly died. A forensic autopsy was performed at 7 days after death. The macroscopic examination revealed pulmonary edema and bilateral adrenal hemorrhage. During the autopsy, namely before internal examination, blood (from neck vessels), and cerebrospinal fluid (through lumbar puncture) samples were collected with sterile techniques for subsequent microbiological examinations. The methods here previously mentioned were used. *N. meningitidis* was found to grow in cultures of blood and cerebrospinal fluid samples after incubation for 48 h, thus providing the postmortem diagnosis of sepsis due to *N. meningitidis*. A premortem diagnosis was not possible because the fulminant disease caused the baby to die very quickly. Indeed, in this second case, the detection of *N. meningitidis* in postmortem cultures was crucial for the determination of the cause of the death. However, the most remarkable finding was the detection of *N. meningitidis* in cultures of samples preserved for 7 days after the death of the patient.

To the best of our knowledge, there is only one other case of late postmortem detection of *N. meningitidis* reported in the literature. In this 2013 case report, group B *N. meningitidis* was identified in a putrefied corpse of a man who was found dead at home (Maujean et al., 2013). The last time that this man had been seen alive was about 10 days previously. No other information regarding the time of death was available.

Formerly, the persistence of *N. meningitidis* in corpses has been identified only after a few hours after death, between 4 and 10 h (Ploy et al., 2005). In contrast, the observations presented here suggest that *N. meningitidis* can survive for more than 10 days after the death of an infected subject. The factors that can contribute to the growth of the bacteria in corpses are various. The abundance of nutritional elements like iron, amino acids, and other carbon sources is likely to be of importance, in combination with the absence of host defenses in corpses (Zughaier et al., 2014).

However, these findings indicate that pathologists as well as other mortuary workers should exercise special precautions when working with corpses infected with *N. meningitidis* because the biological fluids and tissues of the corpses are still infectious for many days after death.

We believe that these two reports are remarkable because they improve the current knowledge regarding infections due to *N. meningitidis*, an extremely relevant pathogen that causes a high mortality rate, especially among young people.

## Author contributions

AO performed one of the autopsies, collected the data, and critically reviewed the manuscript. SV participate in the data collection and drafted the manuscript. AC performed the microbiological examinations and critically reviewed the manuscript. PB performed the microbiological examinations and critically reviewed the manuscript. LA collected the data and performed one of the autopsies. AT critically reviewed the manuscript.

### Conflict of interest statement

The authors declare that the research was conducted in the absence of any commercial or financial relationships that could be construed as a potential conflict of interest.
